# Reverse transcription-recombinase-aided amplification and CRISPR/Cas12a-based visual detection of maize chlorotic mottle virus

**DOI:** 10.1186/s42483-022-00128-y

**Published:** 2022-06-20

**Authors:** Xueyan Duan, Wendi Ma, Zhiyuan Jiao, Yiying Tian, Ragab Gomaa Ismail, Tao Zhou, Zaifeng Fan

**Affiliations:** 1 State Key Laboratory of Agrobiotechnology, MARA Key Laboratory of Surveillance and Management for Plant Quarantine Pests, College of Plant Protection, China Agricultural University, Beijing, 100193 China; 2 Sanya Institute of China Agricultural University, Building 8, Yongyou Industrial Park, Yazhou Bay Science and Technology City, Yazhou District, Sanya, 572025 Hainan China; 3 Department of Plant Pathology, College of Agriculture, Alexandria University, El-Shatby, Alexandria, 21545 Egypt

**Keywords:** MCMV, CRISPR/Cas12a, RT-RAA, On-site detection

## Abstract

Maize chlorotic mottle virus (MCMV) is one of the important quarantine pathogens in China. It often co-infects with one or two viruses in the family *Potyviridae* and causes maize lethal necrosis disease. Therefore, an accurate and sensitive method for the detection of MCMV is urgently needed. Combined with reverse transcription and recombinase-aided amplification, we developed a CRISPR**/**Cas12a-based visual nucleic acid detection system targeting the MCMV coat protein gene. The whole process can be completed within 45 min with high sensitivity. This system could detect cDNAs diluted up to 10^–5^ when 2000 ng of total RNA was used for reverse transcription. The Cas12a/crRNA complex designed for MCMV detection could recognize and cleave the targeted double-stranded DNA, and ultimately cleave the single-stranded DNA probes and produce fluorescent signals. The green fluorescence produced under blue light (440–460 nm) in this procedure could be observed by the naked eye. Since this novel method is specific, rapid, sensitive and does not require special instruments and technical expertise, it should be suitable for on-site visual detection of MCMV in seeds, plants of maize and potentially in its insect vectors.

## Background

Maize chlorotic mottle virus (MCMV) is the only member of the genus *Machlomovirus* in the family *Tombusviridae* (Scheets 2016). The MCMV genome contains a positive-sense single-stranded RNA of 4437 nucleotides (nts) and encodes seven proteins, i.e. P32, P50, P111, P7a, P7b, P31, and coat protein (CP) (Nutter et al. 1989; Scheets 2016). Since its discovery in Peru in 1971 (Castillo and Hebert 1974), MCMV has caused tremendous damage in many areas around the world, including countries in North America, South America, Asia, Europe, and Africa (Xie et al. 2011; Redinbaugh and Stewart 2018). MCMV can be transmitted by insects, seeds, and mechanical means. Several species of leaf beetles and thrips are the main insect vectors of MCMV (Jensen 1985; Jiang et al. 1992), thus widespread distribution of these insect vectors greatly increases the risk of MCMV transmission. The long-distance transmission of MCMV is caused mainly via seeds infected with the virus (Jensen et al. 1991). MCMV causes mosaic, chlorosis and mottle symptoms, and even necrosis when it infects some varieties of maize (Jiao et al. 2021b, c). The co-infection of MCMV and one or more viruses in the family *Potyviridae*, such as sugarcane mosaic virus (SCMV) (Xia et al. 2016; Wang et al. 2017; Jiao et al. 2022), maize dwarf mosaic virus (MDMV) (Goldberg and Brakke 1987), johnsongrass mosaic virus (JGMV) (Stewart et al. 2017), and wheat streak mosaic virus (WSMV) (Scheets 1998) can cause maize lethal necrosis disease (MLND), leading to considerable yield losses (Redinbaugh and Stewart 2018). MCMV has become a serious threat to maize production in some countries, thus it is now a quarantine virus in China and many other countries or regions.

Rapid detection of a viral causative agent is necessary to prevent its spread. Several methods have been developed for the detection of MCMV, including enzyme-linked immunosorbent assay (ELISA) (Uyemoto 1980), real-time TaqMan RT-PCR (Zhang et al. 2011), next-generation sequencing (Adams et al. 2013), and isothermal detection methods such as reverse transcription-loop-mediated isothermal amplification (RT-LAMP) (Chen et al. 2017), and recombinase polymerase amplification (RPA) (Jiao et al. 2019). However, these developed methods have some limitations in achieving rapid and on-site detection of MCMV. Most currently used detection methods are time-consuming and require expensive equipment and technical expertise, restricting their diagnostic application outside the laboratory. Therefore, it is necessary to establish a rapid, sensitive and user-friendly method for the on-site detection of MCMV.

A novel nucleic acid detection technology has been developed based on CRISPR-associated (Cas) endoribonuclease systems including Cas13a or Cas12a (Gootenberg et al. 2017; Chen et al. 2018). The CRISPR-associated protein Cas12a is an endonuclease of the class 2 CRISPR-Cas system, which recognizes a T-rich protospacer-adjacent motif (PAM) under the guidance of CRISPR RNA (crRNA) (Zetsche et al. 2015). Chen et al. (2018) found that Cas12a could cleave single-stranded DNA (ssDNA) indiscriminately after cleaving the targeted double-stranded DNA (dsDNA). Accordingly, DNA endonuclease-targeted CRISPR trans-reporter (DETECTR) method was developed to detect human papillomavirus (HPV)16 and HPV18 (Chen et al. 2018), and several other visual nucleic acid detection methods have been developed thereafter. A novel coronavirus (SARS-CoV-2) detection based on CRISPR/Cas12a could be completed within 45 min, and the results were visualized by the naked eye under blue light (Wang et al. 2020). Combined with RT-LAMP and CRISPR/Cas12a, SARS-CoV-2 can be detected, and the fluorescence produced could be recorded with a smart phone and portable 3D printing instrument (Chen et al. 2020). A CRISPR/Cas12a-based visual assay for the detection of multiple RNA viruses/viroid in apple was established utilizing oligonucleotide-conjugated gold nanoparticles and linker-ssDNA (Jiao et al. 2021a).

Here, we developed a specific, sensitive, and visual method for the detection of MCMV based on RT-recombinase-aided amplification (RAA) and CRISPR/Cas12a activities. RAA is an isothermal nucleic acid amplification procedure which is similar to RPA, in which recombinase, single-stranded DNA-binding protein (SSB), and DNA polymerase replace the thermal cycle amplification process, and the rapid and efficient amplification can be achieved at 37–39 °C. Combined with RAA, the entire detection process can be completed within 45 min without using any sophisticated equipment and instruments. Once the RAA amplicon is added to CRISPR/Cas12a reaction system containing ssDNA probes, positive targets would activate the ssDNA endonuclease activity of Cas12a to cleave ssDNA probes and produce fluorescent signals which can be visualized under blue light (440–460 nm). Our results demonstrated that this method is specific and sensitive, and should be applicable for the on-site detection of MCMV.

## Results

### Optimization of the conditions for CRISPR/Cas12a-based visual detection

**Fig. 1:**
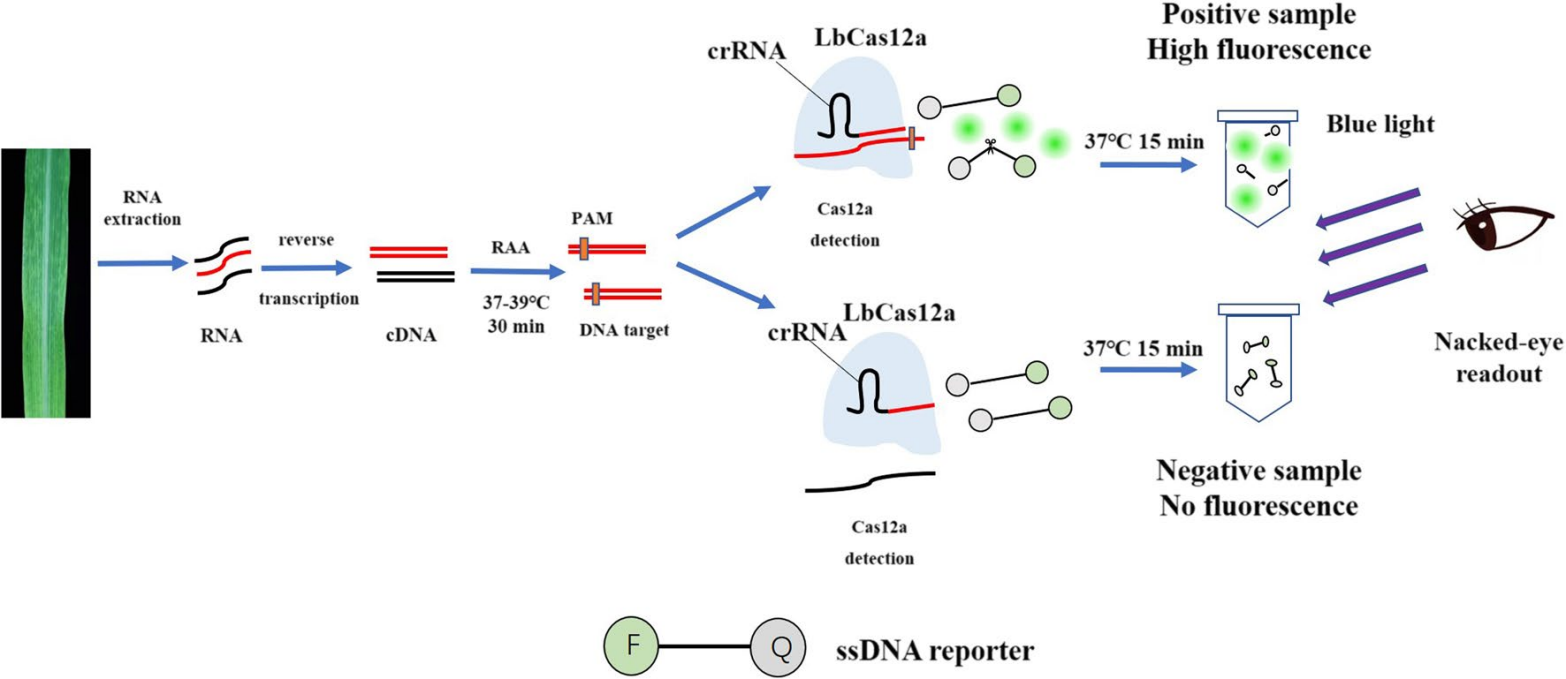
Schematic diagram of the method for rapid and visual nucleic acid detection using RT-RAA-CRISPR/Cas12a assay. In this detection system, target gene fragments were amplified with RAA procedure; ssDNA probes (5′-FAM/3′-BHQ1 labeled) were added, and subsequently cleaved by Cas12a to generate green fluorescence; the detection results were visible to the naked eye under blue light

The RT-RAA-CRISPR/Cas12a-based nucleic acid detection platform is shown in Fig. 1. After amplification, the RAA product was used for detection assay with CRISPR/Cas12a. An ssDNA labeled with a quenched green-fluorescent molecule was introduced to realize visual detection. The ssDNA will be cleaved by Cas12a once the target molecule is present in the system, which activates a significant green fluorescence signal. In contrast, the ssDNA will not be cleaved if the system lacks virus-derived dsDNA, and no fluorescence signal will be produced. The detection results can be observed directly by the naked eye under blue light after reacting for approximately 15 min. The preliminary experiments were conducted with the template target-40 and its crRNA as previously reported (Jiao et al. 2021a).

**Fig. 2:**
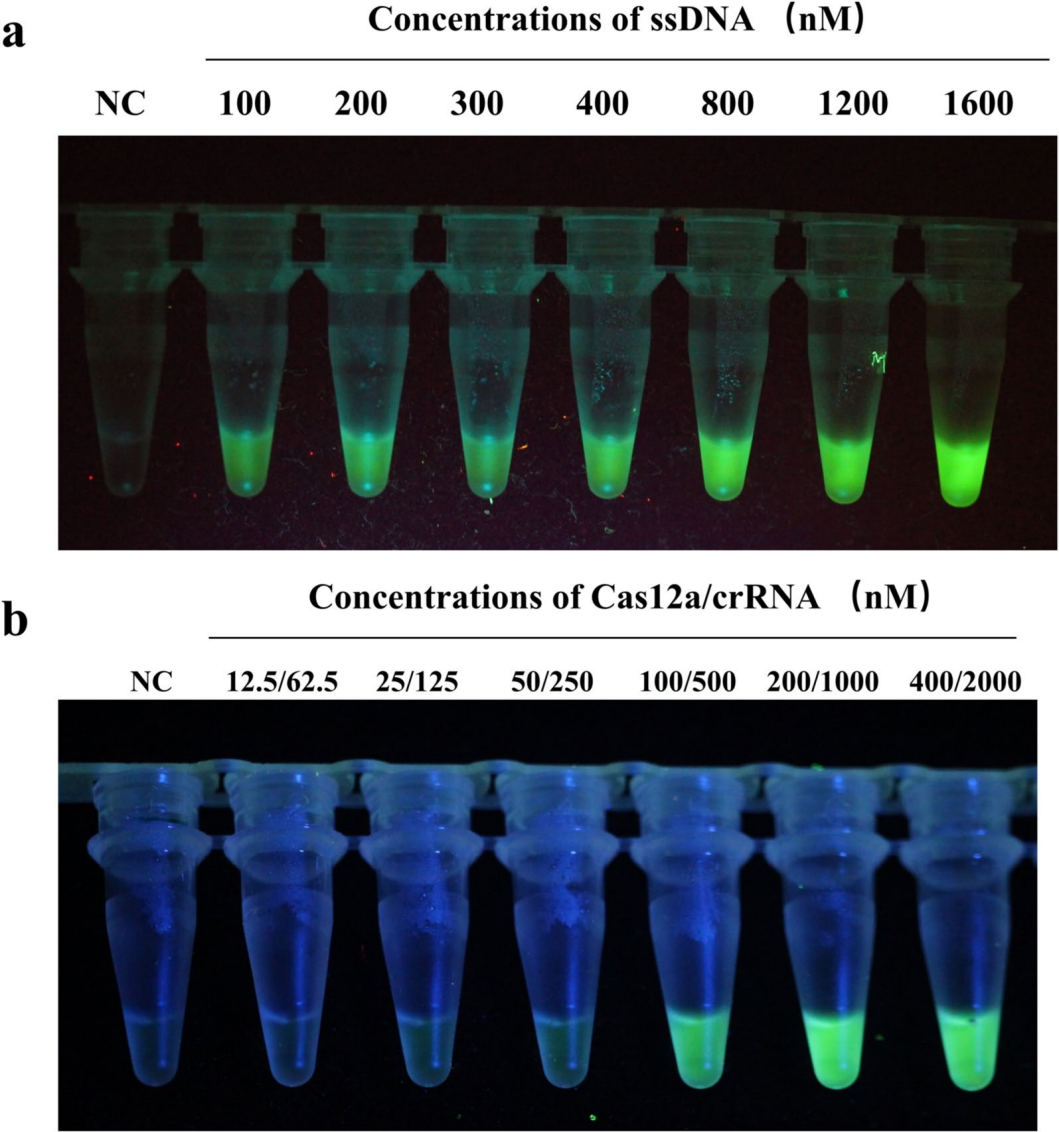
Optimization of the reaction components and conditions for CRISPR/Cas12a-based visual detection. **a** Determination of optimal ssDNA concentration by comparison. **b** Determination of optimal Cas12a/crRNA concentration by visual observation

Here, we first investigated the dosage of ssDNA that can induce obvious green fluorescence. Except for the concentration of ssDNA, the other ingredients and their concentrations in the reaction system were the same. A series of concentrations of ssDNA (0, 100, 200, 300, 400, 800, 1200, and 1600 nM) were tested. As shown in Fig. 2a, clear green fluorescence was produced when the concentration of ssDNA reached 800 nM, and no significant difference in signal intensity was observed with the ssDNA concentration ranging from 800 to 1600 nM. Thus, a final ssDNA concentration of 800 nM was used for further experiments.

After determining the optimal concentration of ssDNA, we subsequently optimized the dosage of LbCas12a and crRNA. The assay was performed with different concentrations of LbCas12a-crRNA complexes as shown in Fig. 2b. When the concentrations of LbCas12a and crRNA were at 200 and 1000 nM, respectively, a clear green fluorescence was observed, with the signal intensity stronger than that produced at a lower concentration. Accordingly, the optimal concentration was determined to be 200 nM for LbCas12a, and 1000 nM for crRNA.

### The developed CRISPR/Cas12a-based visual system is specific for the detection of MCMV

**Fig. 3:**
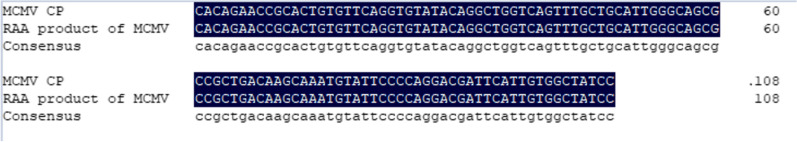
Alignment of the nucleotide sequence of the RAA-amplified product against the *CP* gene sequence of the infectious clone of MCMV (pMCM41). MCMV CP, the partial nucleotide sequence of the *CP* gene of the MCMV full-length cDNA clone (pMCM41); RAA product of MCMV, the partial nucleotide sequence of the RAA-amplified product of MCMV *CP* gene

**Fig. 4:**
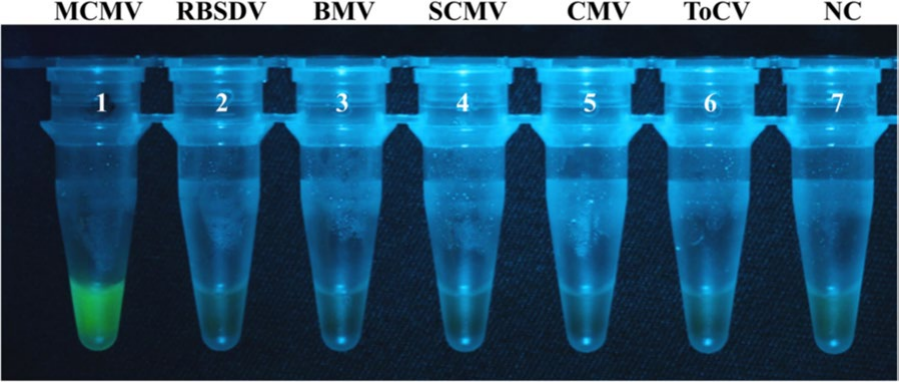
Specificity test of the established RT-RAA-CRISPR/Cas12a detection for MCMV. The tubes 1 to 7 indicate MCMV, RBSDV, BMV, SCMV, CMV, ToCV and ddH_2_O, respectively

The target gene fragments of a virus can be quickly amplified via RAA procedure. The amplification product was purified, sequenced, and compared with the full-length cDNA sequence of an MCMV clone (pMCM41) (Scheets et al. 1993). It was shown that the RAA amplification product was indeed derived from the MCMV *CP* gene (Fig. 3), which demonstrated that the RAA system effectively amplified a fragment of the target gene and the amplification product could be used for the follow-up detection. Subsequently, samples containing one of 5 other viruses (the cDNA of RBSDV, BMV, SCMV, CMV, or ToCV) were used as out-group controls to investigate the specificity of RT-RAA-CRISPR/Cas12a for the detection of MCMV. One reaction without any virus was included as a negative control. The results showed that only the MCMV-derived RAA amplification product generated robust green fluorescence, and there were no significant fluorescence signals for both out-group viruses and negative controls (Fig. 4). This indicates that the RT-RAA-CRISPR/Cas12a visual system developed in this study is specific for the detection of MCMV.

### Sensitivity assay for RT-RAA-CRISPR/Cas12a detection

**Fig. 5:**
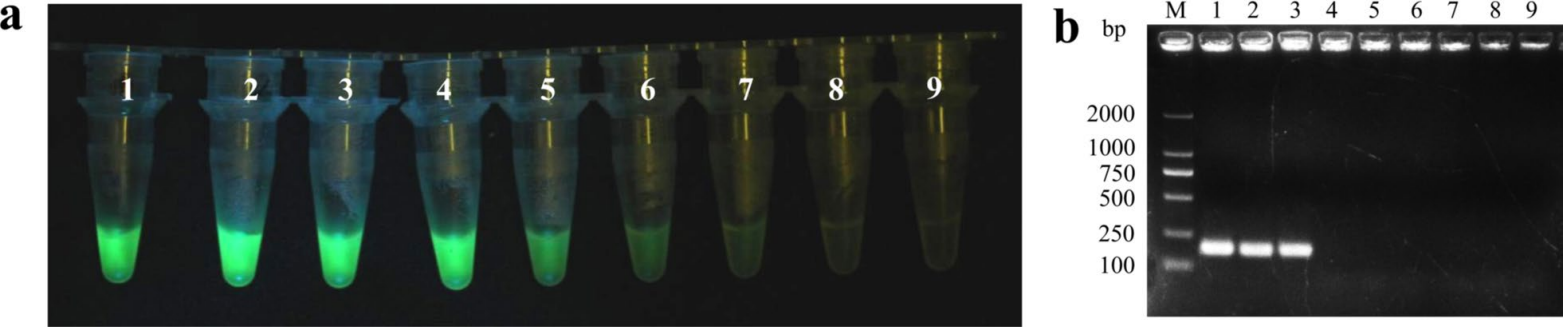
Sensitivity assay for RT-RAA-CRISPR/Cas12a detection. **a** The RAA products (amplicons) from tenfold serial dilutions of MCMV cDNA was applied to RT-RAA-CRISPR/Cas12a detection. Tubes 1–8, tenfold serial dilutions (10^0^–10^–7^) of MCMV cDNA (the reverse transcription reaction was initiated with 2000 ng of total RNA); Tube 9, ddH_2_O as a blank (negative) control. **b** Results of agarose gel electrophoresis of the PCR products. Lane M, DL2000 DNA marker; Lanes 1–8, PCR amplicon from tenfold serial dilutions (10^0^–10^–7^) of MCMV cDNA (the same concentration as in **a**); Lane 9, ddH_2_O as a negative control

To determine the sensitivity of the RT-RAA-CRISPR/Cas12a method, we serially diluted the MCMV cDNA (the reverse transcription reaction was initiated with 2000 ng of total RNA) via tenfold dilutions (10^0^, 10^–1^, 10^–2^, 10^–3^, 10^–4^, 10^–5^, 10^–6^, and 10^–7^), which were used as templates for RAA reaction, with distilled water as a negative control. After reacting for 30 min, the RAA product was applied for CRISPR/Cas12a detection. Green fluorescence could still be observed when the cDNA was diluted up to 10^–5^ (Fig. 5a). To determine the minimal detection limit of the traditional PCR, we conducted PCR assay with the above-mentioned serially diluted MCMV cDNA as templates. The results showed that there was a clear band when the MCMV cDNA was diluted to 10^–2^ (Fig. 5b). This indicates that the sensitivity of the newly developed RT-RAA-CRISPR/Cas12a system is approximately 1000 times higher than that of the traditional RT-PCR method.

## Discussion

Maize diseases caused by MCMV infection are widely distributed worldwide. Rapid and accurate detection of the virus is helpful for preventing the epidemics. However, existing diagnostics for MCMV can be performed only in laboratory. There is a delay between sample collection and acquisition of detection results. In this study, we reported a method for visual detection of MCMV based on CRISPR/LbCas12a system, including RAA reaction and CRISPR/LbCas12a detection. In this detection system, the ssDNA (5′-FAM/3′-BHQ1 labeled) probes were added and could be cleaved by the ssDNase activity of Cas12a to produce green fluorescence. The detection results can be observed directly under blue light, which increases the portability of the method. Therefore, this method has the potential to be used in the field or for on-site detection.

In this study, the primers and crRNAs were designed based on the highly conserved *CP* gene sequence of MCMV. The primers and crRNA with high specificity and efficiency were selected for subsequent detection. The ssDNA probe developed in this research is not only specific to MCMV but also could be used to detect other viruses. For example, we detected southern rice black-streaked dwarf virus (SRBSDV) with the same method and ssDNA probe (unpublished results). The extensive applicability of an ssDNA probe can drastically reduce the cost of this RT-RAA-CRISPR/Cas12a-based detection system.

Compared with other nucleic acid detection technologies, this method shows obvious advantages in sensitivity, reaction time, efficiency, and simplicity. The sensitivity of RT-RAA-CRISPR/Cas12a is *circa* 1000 times higher than that of traditional RT-PCR amplification. Unlike traditional PCR assays, RAA reaction does not require repeated heating and cooling process, because the recombinase can untie dsDNA to start amplification without heating. The CRISPR/LbCas12a reaction can be completed within 15 min. The combination of RAA and CRISPR/LbCas12a method significantly shortens the reaction time, and the whole amplification and visual detection process could be completed within 45 min at *circa* 37 °C. Our results indicate that the method is accurate and reliable. In addition, the entire process does not need any complex instrument and is easy to operate, which makes it not limited to the laboratory. Since the detection system only requires reagents, centrifuges, pipettes, tips, portable movable blue light, heating block, combined with the extraction of nucleic acids from rapidly-prepared sample lysis, it could achieve rapid detection for field samples. Compared with the detection procedure employing Cas12a combined with gold nanoparticles, the RT-RAA-CRISPR/Cas12a system applies portable blue light to observe detection results, eliminating experimental steps such as the preparation of gold nanoparticles. Additionally, the RT-RAA-CRISPR/Cas12a system is superior to the detection method of RT-LAMP combined with Cas12a. LAMP requires longer reaction time and more complex primer designs than RAA and needs a reaction temperature of 65 °C to complete amplification. The primer designing for RAA is simpler and the temperature required is lower compared with other established procedures for MCMV detection. In conclusion, we developed a rapid, specific, and sensitive method for the detection of MCMV. This method is promising for on-site detection of MCMV, which will be conducive to timely disease diagnosis and control of this virus.

## Conclusions

In this study, a rapid, specific, and sensitive method for the visual detection of MCMV has been developed. Specificity verification and sensitivity analysis indicate that this RT-RAA-CRISPR/Cas12a detection system for MCMV is effective, which could detect MCMV specifically and distinguish it from other unrelated plant viruses within 45 min. This detection system for MCMV is highly efficient and its detection limit is significantly lower than that of traditional RT-PCR method.

## Methods

### Preparation of materials

MCMV was prepared from the full-length cDNA clone (pMCM41) (Scheets et al. 1993). Maize leaf samples infected with rice black-streaked dwarf virus (RBSDV), sugarcane mosaic virus (SCMV), cucumber mosaic virus (CMV), brome mosaic virus (BMV), respectively, and *Nicotiana benthamiana* plants infected with tomato chlorosis virus (ToCV) were preserved in our laboratory. RT-PCR with specific primers of each of these viruses was conducted to verify that the samples were indeed infected. RAA primers and ssDNA probes were synthesized by Tsingke Biological Technology (Beijing, China). A 40-bp dsDNA target (target-40) was formed by annealing of two complementary oligonucleotides, NTS-40 (5'-TCACGCTCGTCGTTTGGTATGGCTTCATTCAGCTCCGGTT-3') and TS-40 (5'-AACCGGAGCTGAATGAAGCCATACCAAACGACGAGCGTGA-3') (Jiao et al. 2021a).

### Total RNA extraction and cDNA synthesis

Total RNA was extracted from leaf samples using TRIzol Reagent (Takara, Dalian, China), according to the manufacturer’s protocol. The concentration of RNA extract was quantified with NanoDrop 2000C microvolume UV–vis spectrophotometer (Thermo Fisher Scientific Inc. Waltham, MA). The first strand of cDNA was synthesized with M-MLV reverse transcriptase (Promega, Madison, WI, USA).

### RAA primer design

The coat protein (CP) gene sequences of MCMV isolates from different countries are highly conserved based on the alignment of sequences available in the GenBank database, and a pair of MCMV-specific RAA primers targeting the *CP* gene were designed. A 30-nt forward primer (MCMV-RAA-F: 5'-CTCAGCTACAATAGCTCTGAAGAACAGAGG-3') and a 30-nt reverse primer (MCMV-RAA-R: 5'-TTGTGTTGCACTAGCTTTGGGGATAGCCAC-3') were synthesized and used for RAA amplification, and the length of the predicted amplification product should be 175 bp.

### RAA assay

The RAA assay was performed using the RAA kit (WLB8201KIT, Nanjing Warbio Biotechnology Co., Ltd). The reaction mixture contained 29.4 μL of buffer A, 12.1 μL of nuclease-free water, 2 μL of forward primer (10 μM), 2 μL of reverse primer (10 μM), 2.5 μL of buffer B, and 2 μL of cDNA template. Buffer B was pre-loaded inside the lid, and the reaction tube was centrifuged briefly to ensure the reagents well-mixed. RAA assay was performed at 37–39 °C for 30 min, followed by purification. RAA product (50 μL) and an equal volume of Tris-saturated phenol/chloroform (1:1, V: V) were thoroughly mixed, and centrifuged at 13,800 *g* for 5 min at room temperature. After collection, the supernatant was transferred to a new tube, and the purified RAA amplification product was kept at −20 °C for subsequent reactions.

### PCR assay

The PCR assay was conducted using a 2 × *Taq* PCR Mix-Plus Kit (Lablead, Beijing, China) with the MCMV-specific RAA primer pairs. The reaction system (25 μL) contains 12.5 μL of 2 × reaction mix, 1 μL of DNA template, 0.5 μL of forward primer (MCMV-RAA-F), 0.5 μL of reverse primer (MCMV-RAA-R), and 10.5 μL of ddH_2_O. The procedure of PCR was as follows: predenaturation (94 °C for 5 min), 30 cycles of amplification (94 °C for 30 s, 54 °C for 30 s and 72 °C for 30 s), and 72 °C for 10 min.

### The design of crRNAs

The crRNAs of LbCas12a recognize a 20-nt target sequence adjacent to a 5′-TTTN-3′ site. The crRNAs were designed to recognize the region which is located within the amplicon between the MCMV-specific RAA primer pairs by employing CRISPR-DT-Cpf1 online software; the efficiency of each crRNA was calculated with this software, and the crRNAs with high efficiency were selected for viral detection. The MCMV-crRNA (5'-AAUUUCUACUGUUGUAGAUGGGAUAGCCACAAUGAAUCG-3') was subsequently used for CRISPR/Cas12a detection.

### Synthesis of crRNAs

The DNA templates encoding crRNAs were synthesized by annealing oligonucleotides with T7-crRNA-F (5'-GAAATTAATACGACTCACTATAGGG-3') as described previously (Li et al. 2017). The T7-MCMV-R oligonucleotide (5'-CGATTCATTGTGGCTATCCCATCTACAACAGTAGAAATTCCCTATAGTGA GTCGTATTAATTTC-3′) was annealed with T7-crRNA-F to form a dsDNA, which encodes MCMV-crRNA. T7-T40-R (5'-GGAGCTGAATGAAGCCATACATCTA CAACAGTAGAAATTCCCTATAGTGAGTCGTATTAATTTC-3′) was annealed with T7-crRNA-F to form a dsDNA, which encodes target-40 crRNA (Jiao et al. 2021a). The assay was performed with an equal volume of two paired oligonucleotides (10 μM) in 1 × annealing buffer (Solarbio, Beijing, China) in a 20-µL volume by using a thermocycler (Jiao et al. 2021a). The whole procedure of annealing was denaturation at 95 °C for 2 min, subsequently, allow the temperature to drop for annealing by 1 °C per 90 s, and then stored at −80 °C before use. We used the T7 RiboMAX™ Express Large Scale RNA Production System Kit (Promega, Madison, WI, USA; Cat: P1320) to transcribe crRNAs according to the manufacturer’s protocol. After purification, the concentrations of the crRNAs were quantified by using NanoDrop 2000C microvolume UV–vis spectrophotometer (Thermo Fisher Scientific Inc. Waltham, MA). The crRNAs were stored at −80 °C before use.

### CRISPR/Cas12a-based visual detection of MCMV

The total CRISPR/Cas12a reaction volume was 25 μL, and the reaction mixture contained 200 nM EnGen Lba Cas12a (Cpf1) (New England Biolabs Inc., Ipswich, MA, USA), 1 μM crRNA, 2.5 μL of 10 × NEBuffer 2.1 Reaction Buffer, 20 U of RRI, 2 μL of RAA amplification product, 800 nM fluorophore quencher labeled ssDNA probe (5′-FAM-CCGGAAAAAAAAAAAACCGG-BHQ1-3′). The assay was performed at 37 ^o^C for 15 min. Ultimately, the results were observed and examined directly by the naked eye under blue light (wavelength: 440–460 nm).

## Data Availability

Not applicable.

## Abbreviations

BMV: Brome mosaic virus
Cas: CRISPR-associated
CMV: Cucumber mosaic virus
CP: Coat protein
CRISPR: Clustered regularly interspaced short palindromic repeats
crRNA: CRISPR RNA
dsDNA: Double-stranded DNA
LAMP: Loop-mediated isothermal amplification
MCMV: Maize chlorotic mottle virus
PAM: Protospacer adjacent motif
PCR: Polymerase chain reaction
RAA: Recombinase-aided amplification
RBSDV: Rice black-streaked dwarf virus
RPA: Recombinase polymerase amplification
RT: Reverse transcription
SCMV: Sugarcane mosaic virus
SSB: Single-stranded DNA-binding protein
ssDNA: Single-stranded DNA
